# Supplementary material for: Lactobacillus plantarum 17-1 Ameliorates DSS-Induced Colitis by Modulating the Colonic Microbiota Composition and Metabolome in Mice
Source: Nutrients. 2025 Apr 15;17(8):1348. doi: 10.3390/nu17081348 (PMC12030267; doi:10.3390/nu17081348)
Supplement: Supplementary file 1 [file nutrients-17-01348-s001.zip › nutrients-3552769-supplementary.pdf]

Lactobacillus plantarum 17-1 Ameliorates DSS-Induced Colitis by Modulating the Colonic Microbiota Composition and Metabolome in Mice

Beibei He [1], Tao Duan [1], Dandan Hu [1,2], Lixian Chen [1], Lin Qiao [1], Dan Song [1], Li Wang [1], Shijie Fan [1], Kunru Teng [1], Weiwei Wang [1,*] and Aike Li [1,*]

[1] Academy of National Food and Strategic Reserves Administration, Beijing 100037, China; hbb@ags.ac.cn (B.H.); dt@ags.ac.cn (T.D.); m18855995768@163.com (D.H.); clx@ags.ac.cn (L.C.); ql@ags.ac.cn (L.Q.); sd@ags.ac.cn (D.S.); wl@ags.ac.cn (L.W.); fsj@ags.ac.cn (S.F.); tkr@ags.ac.cn (K.T.)

[2] Faculty of Food Science and Engineering, Central South University of Forestry and Technology, Changsha 410004, China

* Correspondence: www@ags.ac.cn (W.W.); lak@ags.ac.cn (A.L.)

Supplementation Table S1. Differential metabolites between DSS and CON groups annotated in KEGG database.

Metabolite	Regulate	*P*_value	DSS mean	CON mean
5-O-Methylembelin	down	2.32E-05	3.65	5.125
L-Homocystine	down	0.02172	2.86	3.512
L-Homoserine	down	0.0005049	5.173	5.548
Glycocholic acid	down	0.01355	2.085	3.106
Bile acid	down	0.01986	5.18	6.08
7-Oxodeoxycholate	down	0.007854	4.891	5.651
10-Oxodecanoate	down	0.002285	3.644	4.021
D-Aspartic acid	down	0.006694	4.595	4.999
1-Palmitoylglycerol 3-phosphate	down	0.02506	4.386	4.854
3beta,7alpha-Dihydroxy-5-cholestenoate	down	0.01016	4.642	5.048
Murideoxycholic acid	down	0.03971	7.313	7.724
(S)-10,16-Dihydroxyhexadecanoic acid	down	0.003287	4.384	4.64
9,10,18-Trihydroxystearate	down	0.002085	4.888	5.126
alpha-Linolenic acid	down	0.003456	5.192	5.416
4-Maleylacetoacetic acid	up	0.009389	3.803	3.237
3-Ketosphingosine	up	1.94E-05	4.931	4.58
6-Hydroxynicotinate	up	0.003244	3.322	2.97
Cholestenone	up	0.0009164	5.652	5.346
Cellobiose	up	0.01029	5.628	5.353
5-Acetamidopentanoate	up	0.01025	3.918	3.667

Supplementation Table S2. Differential metabolites between DSS and LP + DSS groups annotated in KEGG database.

Metabolite	Regulate	*P*_value	LP + DSS mean	DSS mean
(9Z)-(7S,8S)-Dihydroxyoctadecenoic acid	down	2.23E-14	3.1	4.904
4-Maleylacetoacetic acid	down	0.004617	3.133	3.803
D-(+)-Malic acid	down	0.002096	4.285	4.798
(9Z,11E)-Octadecadienoic acid	down	0.01548	4.14	4.552
Arachidonic acid	down	0.009662	5.199	5.447
Cellobiose	down	0.03399	5.45	5.628
Anandamide	down	0.002329	5.412	5.541
N-Acetyl-L-phenylalanine	up	1.44E-06	5.075	3.542
D-Urobilinogen	up	0.001879	5.197	4.385
Suberic acid	up	7.49E-07	4.904	4.581
Murideoxycholic acid	up	0.01348	7.784	7.313
Ethyl (E,Z)-decadienoate	up	0.006668	4.716	4.359
9,10,13-TriHOME	up	0.001235	5.195	4.997
Ophiobolin A	up	0.001871	4.941	4.727
(9R,10R)-Dihydroxyoctadecanoic acid	up	0.01106	5.083	4.846
(9Z)-(13S)-12,13-Epoxyoctadeca-9,11-dienoic acid	up	0.0003376	5.32	5.148
9,10-DHOME	up	0.004654	5.32	5.123
（9Z,12Z)-(8R)-Hydroxyoctadeca-9,12-dienoic acid	up	0.007733	5.319	5.116
Tetradecanedioic acid	up	0.005156	4.836	4.649
(4Z,7Z,10Z,13Z,16Z,19Z)-Docosahexaenoic acid	up	0.006578	4.568	4.365
Acetyl-DL-Leucine	up	0.04823	4.423	4.2
Ecklonialactone A	up	0.0001425	5.669	5.541
5-O-Methylembelin	up	0.00034	5.652	5.532
3beta-Hydroxy-4beta-methyl-5alpha-cholest-7-ene-4alpha-carboxylate	up	0.002641	5.989	5.855
